# The Influence of Copper and Zinc on Photosynthesis and Phenolic Levels in Basil (*Ocimum basilicum* L.), Borage (*Borago officinalis* L.), Common Nettle (*Urtica dioica* L.) and Peppermint (*Mentha piperita* L.)

**DOI:** 10.3390/ijms25073612

**Published:** 2024-03-23

**Authors:** Dorota Adamczyk-Szabela, Wojciech M. Wolf

**Affiliations:** Faculty of Chemistry, Institute of General and Ecological Chemistry, Technical University of Lodz, Zeromskiego 116, 90-924 Lodz, Poland; wojciech.wolf@p.lodz.pl

**Keywords:** heavy metals, polyphenols, herbs, photosynthesis

## Abstract

This work is aimed at relationships which govern zinc and copper uptake by four popular medicinal herbs: basil (*Ocimum basilicum* L.), borage (Borago officinalis L.), common nettle (*Urtica dioica* L.) and peppermint (*Mentha piperita* L.). They are often grown in soils with significant copper or zinc levels. Herbs were cultivated by a pot method in controlled conditions. Manganese, iron, copper and zinc concentrations were determined by High-Resolution Continuum Source Flame Atomic Absorption Spectrometry. The efficiency of photosynthesis was estimated by measuring the chlorophyll content, water use efficiency, net photosynthesis, intercellular CO_2_, stomatal conductance, and transpiration rate. Phenolic compounds were determined by the Folin–Ciocalteu method. Analysis of variance showed that herbs grown in soil treated with copper exhibited a lower iron content in roots, while manganese behaved in the opposite way. The only exception was borage, where a decrease in the manganese content in roots was observed. Both copper and zinc supplementations increased the total content of phenolics, while the highest increases were observed for common nettle and basil. Peppermint and borage responded less to supplementation. In the majority of samples, zinc and copper did not significantly affect the photosynthesis. Herbal extracts from common nettle and basil had unique antioxidant properties and may be good free radical scavengers.

## 1. Introduction

Heavy metals play a significant role in the growth and development of plants. Nevertheless, either their deficiency or excess can cause disorders in plant growth and development by affecting important physiological processes in plants [1].

The main sources of heavy metal contamination of soil are mining, exhaust emissions, sewage irrigation [2] and the continuously growing chemicalization of agriculture. However, in many places around the world, deficiencies of essential heavy metals are observed in agricultural crops. This phenomenon is often observed in alkaline soils, where the availability of microelements for plants may be quite limited indeed [3].

Zinc (Zn) and copper (Cu) are essential plant micronutrients [4]. Zinc is a structural component of zinc finger proteins and is pivotal for the synthesis of photosynthetic pigments like chlorophyll [5]. Food and Agriculture Organization of the United Nations (FAO) projects on the assessment of the level of microelements in agricultural soils around the world raise the problem of global zinc deficiency in crops [6]. Increasing the soil concentration of zinc in soils with a substantial deficit of this element brings a number of agronomic benefits in plant cultivation and productivity.

Copper acts as an essential cofactor of several enzymes that play key functions in plant cell metabolism including the respiration, photosynthesis, and scavenging of reactive oxidative species (ROS) [7,8]. On the other hand, the redox properties of copper may contribute to its toxicity. Redox cycles between Cu^2+^ and Cu^+^ can increase the production of highly toxic hydroxyl radicals with subsequent damage to cells at the level of lipids, membranes, nucleic acids, proteins and other biomolecules [9]. Copper usually binds to proteins and has the ability to initiate oxidative damage and disrupt important cellular processes [10].

Therefore, significant changes in the content of copper or zinc in the soil are reflected by their respective levels in plant tissues. Above certain concentrations, those elements act as stressors and prompt the production of ROS [11]. In response, plants synthesize phenolic compounds which are effective free radicals scavengers [12]. 

Medicinal herbs and food species are important sources of bioactive compounds. Following the World Health Organization (WHO), almost 80% of populations heavily depend on herbal therapies [13]. In the polluted environment inhabited by people living in stressing conditions, there is a continuous demand for cheap and safe antioxidants with pronounced free radicals scavenging ability. They are represented by phenolic acids, phenolic diterpenes, flavonoids, and essential oils [14]. Plants containing these compounds are widely used in gastronomy, cosmetic industries, perfumery, and the pharmaceutical industry as well as in herbal medicine [15]. Preliminary studies indicate that they respond to heavy metal stress with the diverse magnitudes [16,17].

Basil (*Ocimum basilicum* L.) from the *Lamiaceae* family is a valuable herb that is used in medicine, food processing and cosmetics [18,19]. It contains a significant level of phenolic acids and flavonoid glycosides [20]. Basil has antispasmodic, antidiabetic, antibacterial, antifungal and antioxidant properties [21,22]. Borage (*Borago officinalis* L.) is an annual herb in the flowering plant family *Boraginaceae*. Its medicinal value is highly appreciated by either the contemporary pharmaceutical industry or traditional medicine. It is used as an effective anti-inflammatory agent in the prevention of colds, bronchitis and respiratory infections [23,24]. Moreover, borage lowers blood cholesterol levels and helps to fight digestive and cardiovascular disorders [25,26]. Common nettle (*Urtica dioica* L.) is a spice from the nettle family (*Urticaceae*). It is a medicinal, cosmetic, edible and feed plant [27]. Nettle has antihemorrhagic properties, increases the number of red blood cells, regulates sugar levels and replenishes the deficiencies of vitamins and mineral salts [28]. Peppermint (*Mentha piperita* L.) belongs to the *Lamiaceae* family in the genus *Mentha* [29]. The herbal raw material are leaves (*Menthae piperitae* folium) containing mainly mint oil, ascorbic acid, carotene, rutin, apigenin, betaine, oleanic and ursulic acids. Peppermint is used for gastrointestinal ailments, migraines and upper respiratory tract diseases. It has antibacterial and calming properties [30,31]. The quality of herbs, their extracts and essential oils strongly depends on the plants’ development and their growing conditions [32]. Herb crops require appropriate fertilization, and the number of permissible plant protection products is very limited.

The goal of this work was to describe relationships which govern zinc and copper uptake by four popular medicinal herbs. The plant metabolism was to be assessed by gasometric analysis. Finally, we aimed at cultivation conditions which prompt the synthesis of phenolic compounds. The latter are useful medicinal substances and may be used as plant stress indicators.

## 2. Results

### 2.1. Soil Analysis

The soil used for growing herbs was acidic (pH 5.3) and belonged to organic soils (86%). The concentrations of heavy metals in this soil did not exceed the limit values (Table 1) in agreement with the international standards [33,34].

### 2.2. Plants Analysis

#### 2.2.1. Heavy Metals Uptake by Herbs

Heavy metal concentrations in the above-ground parts and roots of herbs are presented in Table 2 and Table 3. Iron, copper and zinc accumulated in the roots of all cultivated plants, while manganese accumulated mainly in the above-ground parts. The only exception is peppermint. To assess the impact of all applied treatments on the concentration of heavy metals in cultivated herbs, one-way ANOVA analysis was used at the probability level *p* = 0.05 (Table 4). Calculations showed that both copper and zinc influence the content of manganese and iron in all tested plants. Copper supplementation triggers a decrease in the manganese content in the above-ground parts of borage, common nettle and peppermint. The exception was basil, where an increase in the content of the latter element was observed. It is notable that the addition of copper limited the iron levels in the above-ground parts of all herbs. Zinc supplementation decreased the manganese and iron contents in the above-ground parts of herbs. The ANOVA analysis (Table 5) clearly showed that herbs which had been grown in the soil treated with copper exhibited a lower iron content in roots. Manganese behaved in the opposite way. The only exception was borage, where a decrease in the manganese content in the roots was observed.

#### 2.2.2. Photosynthesis Parameters

**Figure 1:**
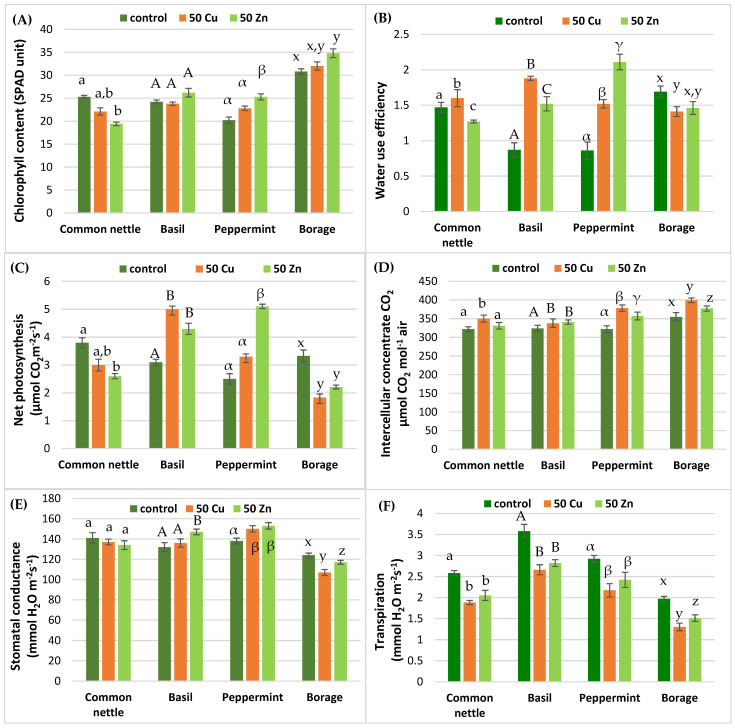
The chlorophyll content (**A**), water use efficiency (**B**), net photosynthesis (**C**), intercellular concentration CO_2_ (**D**), stomatal conductance (**E**), transpiration (**F**). Specific letters illustrate the statistically significant differences as computed with Tukey’s HSD test. Error bars correspond to the S.D. The number of samples *n* = 5, probability level *p* = 0.05.

**Figure 2:**
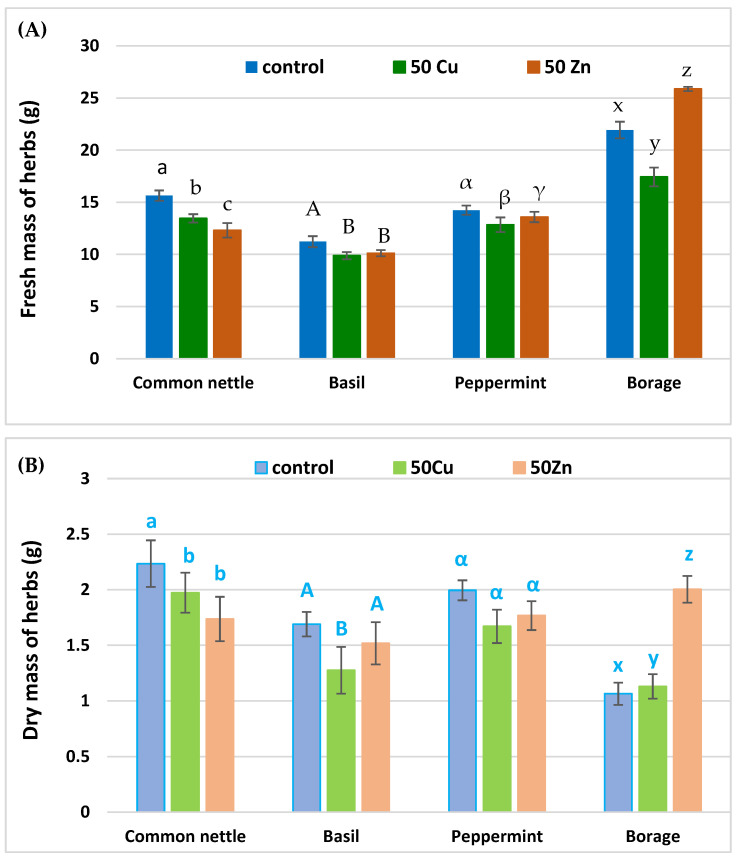
The fresh mass of herbs (**A**) and dry mass of herbs (**B**) cultivated on soil with additives. Specific letters demonstrate the statistically significant differences as computed with Tukey’s HSD test. Error bars correspond to the S.D. The number of samples *n* = 5, probability level *p* = 0.05.

The metabolism of herbs was estimated using photosynthesis indicators, i.e., the index of chlorophyll content in leaves, water use efficiency (WUE), net photosynthesis activity, stomatal conductance, transpiration rate and intercellular CO_2_ concentration (Figure 1). Those parameters clearly showed that herbs were in reasonable growth conditions. The influence of copper or zinc on the chlorophyll content in herbs varies greatly and was dependent on the plant species. A clear decrease was observed only for the common nettle. In peppermint and borage grown in soil with the addition of metals, the content chlorophyll increased. The WUE clearly showed that the analyzed herbs reacted on metals introduced into the soil in quite different ways. The photosynthesis parameters of the analyzed herbs treated with copper or zinc were strictly dependent on the plant species. A common nettle grown on soil supplemented with copper or zinc showed a decrease in the intensity of the photosynthesis process. The opposite situation was observed for peppermint. Basil and borage behaved in a more diverse way. An increase in intercellular CO_2_ content was observed in all analyzed herbs after supplementation with copper and zinc. The mass of common nettle, peppermint and basil-treated zinc or copper (Figure 2) decreased slightly. The only exception was borage grown in soil supplemented with zinc.

#### 2.2.3. Total Phenolic Compounds Content

**Figure 3:**
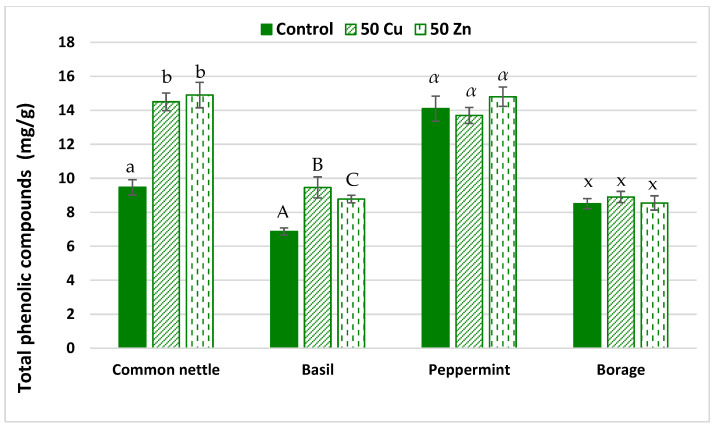
The total phenolic compounds in herbs cultivated in soil with copper and zinc supplementation. Specific letters demonstrate the statistically significant differences as computed with Tukey’s HSD test. Error bars correspond to the S.D. The number of samples *n* = 5, probability level *p* = 0.05.

Figure 3 presents the total phenolic compounds (TPCs) content as determined by the Folin–Ciocalteu method. Both copper and zinc supplementation increased the total content of phenolic compounds in stinging nettle and basil.

The content of phenolic compounds in common nettle increased by 53% after copper supplementation and by 57% when the zinc treatment was applied. For basil, those values were 38% (copper supplementation) and 28% (zinc supplementation), respectively, while no significant differences were observed for peppermint and borage.

## 3. Discussion

It is well known that heavy metals in the soil environment tend to migrate to the rhizosphere and are subjected to the uptake by plant roots there. Subsequently, they are transported via xylem and phloem to the upper parts of the plant [35]. Our investigations clearly show that the impact of heavy metals on herbs is quite diverse and heavily depends on individual species. Significant antagonistic interactions between zinc and copper were observed in all of the herbs investigated. In particular, the zinc uptake could be inhibited by the increased copper content in roots. This result may indicate that the similar carrier sites are involved in the absorption and transport mechanisms of both metals [36].

In particular, Behtash et al. [37] examined the effect of copper and zinc on the morphophysiological characteristics of spring squash. Increased zinc content was observed in the presence of small copper concentrations, while the low zinc levels prompted the copper uptake. The authors pointed out that copper in high concentrations directly had affected the reaction centers of the photosystem and had interfered with photosynthetic processes by annihilating the photosynthetic pigments, membrane stability and photosynthetic enzymes. 

It is common knowledge that any disturbances in the photosynthesis process adversely affect the quality of crops. For this reason, the antagonistic interaction of zinc and copper may significantly reduce the effects of copper toxicity. However, we did not observe this effect along the applied copper supplementation. Zinc contents in the aerial parts of cultivated herbs varied greatly depending on the plant species [37]. The diverse interactions of copper and zinc have been described many times. Le et al. [38] examined the interaction of both those elements and found that the presence of zinc reduces the toxicity of copper, while the presence of copper does not affect the toxicity of zinc. Moreover, results may be biased by interactions with other ions, e.g., zinc is transported through root cells by the ZIP family of transporters, which can also bind iron [39,40,41].

Kabata-Pendias and Pendias [42] report that zinc interferes more with the uptake and transport of iron than copper. This is likely due to competition between Zn^2+^ and Fe^2+^ ions during uptake by plant roots and interference in chelation processes. High levels of copper in the plant decrease the iron content. The optimal copper–iron ratio varies for different plant species. In particular, the uptake and transport of iron are greatly influenced by the concentrations and proportions of other heavy metals [43]. Iron is considered an element that plays a key role in the mechanisms of photosynthesis [44]. 

It is quite common knowledge that heavy metals induce stress to plants and prompt multidirectional metabolic disorders which further limit photosynthesis and biomass productivity. Thus, the reduced weight of plants grown in the soil with increased copper or zinc levels may result from oxidative stress, a limited absorption of essential elements and reduced metabolism [45]. Nevertheless, it should be remembered that the response of plants to stress is closely related to plant species and the level of heavy metals in the cultivation environment. Our research clearly shows that common nettle is the most sensitive to the presence of these additives. However, modifications of the root system may also affect the final weight of particular herbs [46].

Heavy metals may limit the uptake of nutrients, the content of photosynthetic pigments, enzymatic activity and protein biosynthesis [47]. However, several studies indicate that the plant stress induced by toxic elements may induce secondary metabolism [48,49]. The plant response to stress caused by heavy metals affects their stomata. The latter are specialized pores in the epidermis of plant cells involved in the process of photosynthesis, respiration and transpiration. According to the literature data, the exposure of plants to heavy metals may cause damage to the structure and function of stomata and ultimately lead to changes in the physiology and ecology of plants [50]. The reasons for stomatal closure in plants are complex and partially may be related to the concentration of heavy metals and the duration of this contamination [51,52]. Moreover, heavy metals may affect the carbon dioxide concentrations in the plant tissues [53]. In response, plants may increase stomatal conductance to meet their respiratory needs. In addition, the accumulation of heavy metals can lead to the leakage of potassium ions from the plant, negatively affecting the plant’s ability to regulate stomatal closure [54]. We clearly observed this phenomenon in basil and peppermint after supplementation with copper and zinc (Figure 1). In all of the analyzed herbs grown in soil with the addition of metals, an increase in intercellular CO_2_ content was observed.

Physiological processes related to water absorption and nutrient uptake significantly influence the growth and development of plants [55,56]. The literature data [57] clearly show that a significant increase in the content of heavy metals in the cultivation media reduces the water content in plant organs. However, particular plant species use specific mechanisms to maintain plant water balance [58]. Our results showed clearly that WUE values changed substantially for each cultivated plant after the copper or zinc addition.

Upon uptake, heavy metals penetrating plant tissues prompt an increase in reactive oxygen species (ROS), which further lead to redox balance disturbance [59,60]. Increased levels of superoxide anion (O^2−^) and hydrogen peroxide (H_2_O_2_) cause a further peroxidation of membrane lipids and the destruction of oxidative cells [61]. ROS are typically produced in plant cell chloroplasts, mitochondria, and subcellular structures [62]. In response to stress, plants may adapt to it or reduce the ROS level by changing the activity of antioxidant enzymes in their tissues [63,64]. Low levels of ROS can activate plant defense reactions, which leads to an increased activity of antioxidant enzymes. However, when ROS levels exceed the ability of the antioxidant defense system, oxidative damage occurs [65]. 

Polyphenols are compounds naturally existing in plants, and they are involved in natural plant antioxidant activity. They are scavengers of free radicals either in plant or human body environments [66]. Our results clearly showed that the addition of copper or zinc to the soil increased the total content of phenolic compounds in common nettle and basil. However, they responded to applied supplementations in diverse ways. It is well documented that plants grown in contaminated soils react to stress conditions by synthetizing phenolic compounds [66,67,68]. Moreover, Mleczek et al. [41] determined that only high levels of copper and zinc prompted a significant increase in the total phenolics. 

## 4. Materials and Methods

### 4.1. Soil Analysis and Preparation for Cultivation

The soil used to grow herbs was taken from the agricultural area located in Lagiewniki, Poland, according to standards [69] The soil was dried and passed through a 2 mm stainless steel sieve. The potentiometric method was used in pH determination [70]. The content of organic matter was estimated by the gravimetric method [71]. The bioavailable forms of metals were analyzed in 0.5 mol/L HCl extracts. Pseudo-total metals contents in soil were determined in solution obtained by microwave decomposition in a mixed HNO_3_ (65%) and HCl (36%). The Anton Paar Multiwave 3000 (Graz, Austria) apparatus was used. Metal concentrations were determined by the High Resolution-Continuum Source Flame Atomic Absorption Spectrometry (HR-CS FAAS) with the Analytik Jena ContrAA 300 (Jena, Germany) apparatus. Each sample was analyzed five times. 

The air-dried soil was weighed (200 g) into plastic containers. The first series of soil without added metals was a reference (Control). The remaining two series were supplemented with solution of zinc or copper in the form of Cu(NO_3_)_2_ and Zn(NO_3_)_2_. The metal concentrations in these samples were increased by 50 µg/g Cu (50Cu) or 50 µg/g Zn (50Zn), respectively. Copper and zinc supplementations were calculated to be consistent with the amounts of those elements which are being introduced to soil with fertilizers used in herbal agriculture.

### 4.2. Plant Material Preparation

Four herbs were cultivated, i.e., basil, borage, common nettle, and peppermint. Each plant was grown in three series of five replicates (control, samples with the addition of 50 µg/g Cu (50Cu) and samples with the addition of 50 µg/g Zn (50Zn)). The seeds of all plants came from the P.H. Legutko company. Herbs were cultivated in a greenhouse under controlled conditions: temperatures 23 ± 2 and 16 ± 2 °C for day and night, respectively; the relative humidity was limited to 70–75%; the photosynthetic active radiation (PAR) during the 16 h photoperiod was restricted to 400 μmol/m^2^ s. Plant cultivation was carried out for three months. After determining the parameters of photosynthesis, all plants were cut, dried in the air and homogenized.

### 4.3. Photosynthesis Parameters

All measurements were collected five times from plants from each pot. The content of chlorophyll in leaves was measured by Konica Minolta SPAD-502 (Tokyo, Japan) in the red and the near-infrared regions. The activity of net photosynthesis (P_N_), the stomatal conductance (G_S_), the intercellular concentration of carbon dioxide (C_i_), and the transpiration (E) were measured with the gas analyzer TPS-2 (Portable Photosynthesis System, Amesbury MA, USA).

### 4.4. Determination of Total Phenolic Compounds in Herbs by Folin–Ciocalteu Method

The commonly used Folin–Ciocalteu method with gallic acid as the standard was used to measure the total phenolics content in herbs [72]. The boiling water (50 mL) was used to extract dried herbs (1 g). The absorbance measurement was made at a wavelength of 765 nm on the Specol 11, Carl Zeiss (Jena, Germany) instrument.

### 4.5. Determination of Heavy Metals in Herbs

The metals in roots and above-ground parts of herbs contents were measured in mineralizates of plant. The same protocol as used for soil analysis was applied.

The quality assurance and quality control (QA/QC) of metals in plant samples were assessed by determining the metal content in the INCT-MPH-2 certified reference material containing a mixture of selected Polish herbs (Table S1).

### 4.6. Data Analysis

All parameters were determined in parallel for five independent samples. The statistical analysis was made separately for each herb. Bartlett and Hartley tests were used to check the equality of variances (STATISTICA 10 PL package). The normality of the data sets was assessed using the Shapiro–Wilk test [73,74]. The Tukey HDS post hoc test was used to assess statistically significant differences among individual parameters. One-way ANOVA was used to identify significant differences in manganese, iron, copper and zinc contents in the above-ground parts and roots of herbs cultivated in soil with copper and zinc supplementations. 

## 5. Conclusions

The addition of copper or zinc to the soil increased the polyphenol content in common nettle and basil. However, this influence varied substantially depending on the plant species. In most plant samples, it did not significantly affect the photosynthesis. Herbal extracts from common nettle and basil had unique antioxidant properties and may be good free radical scavengers. Investigations on increasing the content of polyphenols in plants are important for their common medical use. On the other hand, determining the safe levels of metals supplemented to the soil in order to increase phenolic compounds in herbs is crucial to obtain proper pharmaceutical materials. 

## Figures and Tables

**Table 1:** Heavy metals content in the soil used in the work. The number of samples *n* = 5, probability level *p* = 0.05.

Metals	Bioavailable Forms (µg/g)	Pseudo-Total Forms (µg/g)	Limit Values (µg/g) *
Mn	75.7 ± 1.0	108 ± 0.6	not applicable
Fe	1323 ± 46	2035 ± 190	not applicable
Cu	9.46 ± 0.06	16.7 ± 0.1	150
Zn	61.8 ± 0.8	93.3 ± 0.39	300

* according to [33,34].

**Table 2:** Heavy metals contents in above-ground parts of herbs cultivated in soils under copper and zinc supplementations. The number of samples *n* = 5, probability level *p* = 0.05. 50Cu = 50 µg/g Cu; 50Zn = 50 µg/g Zn. Specific pairs of letters as given in parentheses illustrate the statistically significant differences between treatments as computed with the Tukey’s HSD test for separate metal and plant combinations.

Treatments	Metal Contents in Above-Ground Parts (µg/g)			
	Mn	Fe	Cu	Zn
**Basil**				
Control	59.7 ± 3.1 (aa)	148 ± 11 (ba)	10.8 ± 1.8 (ca)	73 ± 5 (da)
50Cu	66.3 ± 4.7 (aa)	121 ± 6 (bb)	47.2 ± 3.1 (cb)	52 ± 7 (db)
50Zn	45.1 ± 3.9 (ab)	104 ± 7 (bc)	7.61 ± 1.22 (ca)	191 ± 10 (dc)
**Borage**				
Control	52.8 ± 2.5 (ea)	80.4 ± 6.2 (fa)	10.1 ± 0.8 (ga)	56.7 ± 4.8 (ha)
50Cu	29.8 ± 2.1 (eb)	71.2 ± 5.1 (fb)	29.4 ± 1.9 (gb)	60.2 ± 5.0 (hb)
50Zn	30.6 ± 2.1 (eb)	65.4 ± 6.8 (fc)	8.53 ± 0.88 (gc)	138 ± 8 (hc)
**Common nettle**				
Control	69.7 ± 6.7 (ia)	103 ± 6 (ja)	7.42 ± 0.44 (ka)	29.1 ± 2.8 (la)
50Cu	58.2 ± 7.1 (ib)	95 ± 7 (jb)	28.5 ± 1.9 (kb)	33.8 ± 2.8 (la)
50Zn	47.5 ± 6.3 (ic)	101 ± 7 (ja,jb)	8.12 ± 0.73 (ka)	94.6 ± 9.1 (lb)
**Peppermint**				
Control	63.3 ± 3.1 (ma)	115 ± 11 (na)	7.35 ± 0.72 (oa)	48.6 ± 3.7 (pa)
50Cu	49.6 ± 3.7 (mb)	108 ± 9 (na,nb)	31.8 ± 5.8 (ob)	34.2 ± 3.3 (pb)
50Zn	33.7 ± 2.9 (mc)	92.6 ± 7 (nb)	6.39 ± 2.52 (oa)	105 ± 5 (pc)

**Table 3:** Heavy metals content in roots of herbs cultivated in soils under copper and zinc supplementation. The number of samples *n* = 5, probability level *p* = 0.05. 50Cu = 50 µg/g Cu; 50Zn = 50 µg/g Zn. Specific pairs of letters as given in parentheses illustrate the statistically significant differences between treatments as computed with Tukey’s HSD test for separate metal and plant combinations.

Treatments	Metal Content in Roots (µg/g)			
	Mn	Fe	Cu	Zn
**Basil**				
Control	31.2 ± 2.1 (aa)	198 ± 6 (ba)	15.9 ± 1.9 (ca)	109 ± 6 (da)
50Cu	70.5 ± 4.3 (ab)	181 ± 5 (bb)	113 ± 5 (cb)	92.1 ± 7.2 (db)
50Zn	17.1 ± 3.4 (ac)	138 ± 7 (bc)	13.2 ± 2.0 (cd)	287 ± 12 (dc)
**Borage**				
Control	40.8 ± 2.5 (ea)	114 ± 6 (fa)	11.5 ± 0.8 (ga)	62.7 ± 4.8 (ha)
50Cu	32.4 ± 2.1 (eb)	85.8 ± 5.1 (fb)	37.4 ± 1.9 (gb)	70.2 ± 5.0 (hb)
50Zn	40.6 ± 1.9 (ea)	75.4 ± 6.8 (fc)	10.1 ± 0.9 (gc)	154 ± 8 (hc)
**Common nettle**				
Control	49.7 ± 6.7 (ia)	172 ± 6 (ja)	9.42 ± 0.44 (ka)	31.1 ± 2.8 (la)
50Cu	68.2 ± 7.1 (ib)	125 ± 7 (jb)	28.5 ± 1.9 (kb)	23.8 ± 2.8 (lb)
50Zn	57.5 ± 6.3 (ic)	137 ± 7 (jc)	7.12 ± 0.73 (kc)	114 ± 9 (lc)
**Peppermint**				
Control	71.3 ± 7.1 (ma)	175 ± 9 (na)	8.34 ± 0.78 (oa)	63.6 ± 7.7 (pa)
50Cu	91.6 ± 8.7 (mb)	128 ± 7 (nb)	43.8 ± 5.8 (ob)	44.2 ± 3.3 (pb)
50Zn	83.7 ± 2.9 (mc)	142 ± 8 (nc)	9.32 ± 2.56 (oa)	115 ± 6 (pc)

**Table 4:** The one-way ANOVA for manganese, iron, copper, and zinc contents in above-ground parts of herbs cultivated in soil under copper and zinc supplementations. Critical Snedecor’s F value is F_cryt_ = 3.8853, probability level *p* = 0.05.

	Basil	Borage	Common Nettle	Peppermint
	Above-Ground Parts			
Mn	*p* = 3.85 × 10^−6^ F = 41.9357	*p* = 1.47 × 10^−8^ F = 115.2513	*p* = 4.95 × 10^−8^ F = 93.0262	*p* = 1.70 × 10^−9^ F = 167.6868
Fe	*p* = 1.21 × 10^−8^ F = 119.1513	*p* = 2.48 × 10^−7^ F = 69.7012	*p* = 2.02 × 10^−2^ F = 5.4899	*p* = 2.12 × 10^−3^ F = 10.7391
Cu	*p* = 6.45 × 10^−11^ F = 387.6501	*p* = 1.52 × 10^−9^ F = 204.5946	*p* = 5.19 × 10^−10^ F = 254.4199	*p* = 5.04 × 10^−11^ F = 407.4172
Zn	*p* = 1.17 × 10^−11^ F = 546.103	*p* = 3.21 × 10^−9^ F = 175.8112	*p* = 1.60 × 10^−10^ F = 322.7082	*p* = 1.11 × 10^−9^ F = 218.2207

**Table 5:** The one-way ANOVA for manganese, iron, copper, and zinc contents in roots of herbs cultivated in soil under copper and zinc supplementations. Critical Snedecor’s F value is F_cryt_ = 3.8853, probability level *p* = 0.05.

	Basil	Borage	Common Nettle	Peppermint
	Roots			
Mn	*p* = 8.23 × 10^−13^ F = 613.7908	*p* = 2.92 × 10^−4^ F = 17.3007	*p* = 1.31 × 10^−7^ F = 78.1844	*p* = 5.46 × 10^−7^ F = 60.3707
Fe	*p* = 7.51 × 10^−9^ F = 129.5797	*p* = 2.69 × 10^−8^ F = 103.6419	*p* = 1.14 × 10^−9^ F = 179.6911	*p* = 8.17 × 10^−8^ F = 85.0893
Cu	*p* = 5.49 × 10^−13^ F = 1010.294	*p* = 3.28 × 10^−8^ F = 109.3599	*p* = 4.32 × 10^−10^ F = 264.0011	*p* = 1.04 × 10^−7^ F = 86.1670
Zn	*p* = 5.72 × 10^−11^ F = 397.1455	*p* = 4.88 × 10^−11^ F = 409.9500	*p* = 7.27 × 10^−9^ F = 148.8850	*p* = 1.17 × 10^−7^ F = 84.1723

## Data Availability

The data sets used and analyzed during the current study available from the corresponding author on reasonable request.

## Supplementary Materials

The supporting information can be downloaded at https://www.mdpi.com/article/10.3390/ijms25073612/s1.
