# Supplementary material for: The Influence of Copper and Zinc on Photosynthesis and Phenolic Levels in Basil (Ocimum basilicum L.), Borage (Borago officinalis L.), Common Nettle (Urtica dioica L.) and Peppermint (Mentha piperita L.)
Source: Int J Mol Sci. 2024 Mar 23;25(7):3612. doi: 10.3390/ijms25073612 (PMC11011574; doi:10.3390/ijms25073612)
Supplement: Supplementary file 1 [file ijms-25-03612-s001.zip › ijms-2875950-supplementary.pdf]

The Influence of Copper and Zinc on Photosynthesis and Phenolic Levels in Basil (*Ocimum basilicum* L.), Borage (*Borago officinalis* L.), Common Nettle (*Urtica dioica* L.) and Peppermint (*Mentha piperita* L.)

Dorota Adamczyk-Szabela [1,*], Wojciech M. Wolf [1]

[1] *Faculty of Chemistry, Institute of General and Ecological Chemistry, Technical University of Lodz, Zeromskiego 116, 90-924 Lodz, Poland*

Corresponding author: Adamczyk-Szabela Dorota, e-mail: dorota.adamczyk@p.lodz.pl

Table S1. Metals concentrations in the certified reference material ($\bar{x} \pm ts_{\bar{x}}$; p = 0.95, n = 5).

Metal	Certified value µg/g	Found µg/g	Recovery %
**Manganese**	191 ± 12	194 ± 9	102
**Copper**	7.77 ± 0.53	7.58 ± 0.51	98
**Zinc**	33.5 ± 2.1	32.8 ± 1.8	98
